# I Am Right, You Are Wrong: How Biased Assimilation Increases the Perceived Gap between Believers and Skeptics of Violent Video Game Effects

**DOI:** 10.1371/journal.pone.0093440

**Published:** 2014-04-10

**Authors:** Tobias Greitemeyer

**Affiliations:** Department of Psychology, University of Innsbruck, Innsbruck, Austria; Brock University, Canada

## Abstract

**Background:**

Despite hundreds of studies, there is continuing debate about the extent to which violent video games increase aggression. Believers argue that playing violent video games increases aggression, but this stance is disputed by skeptics. The present study addressed believers' and skeptics' responses to summaries of scientific studies that do or do not present evidence for increased aggression after violent video game play.

**Methods/Principal Findings:**

Participants (*N* = 662) indicated whether they believed that violent video games increase aggression. Afterwards, they evaluated two opposing summaries of fictitious studies on the effects of violent video play. They also reported whether their initial belief had changed after reading the two summaries and indicated again whether they believed that violent video games increase aggression. Results showed that believers evaluated the study showing an effect more favorably than a study showing no effect, whereas the opposite was observed for skeptics. Moreover, both believers and skeptics reported to become more convinced of their initial view. In contrast, for actual attitude change, a depolarization effect was found.

**Conclusions/Significance:**

These results suggest that biased assimilation of new information leads believers and skeptics to become more rather than less certain of their views. Hence, even when confronted with mixed and inconclusive evidence, the perceived gap between both sides of the argument increases.

## Introduction

The question of whether playing violent video games causes aggression has received considerable attention in scientific and public debate. This is not surprising insofar as video game play has become an integral part of the lives of many people. In the United States, 91% of children between the age of two and seventeen play video games [1]. Another survey found that about 97% of American teens play video games [2]. But video games are not only popular among children. The average age of a video game player is around 35 years [3]. Content analyses of video games showed that most of the popular video games contain violence [4], [5] and surveys revealed that violent video games are highly popular across different age groups [2].

Numerous studies have shown that playing violent video games is associated with increased aggression. Correlational work revealed positive correlations between violent video game play and aggression in real world contexts [6], [7]. Longitudinal investigations found that habitual violent video game play predicts aggression in the long-term. Moreover, the effects of violent video games on aggression remain significant even after controlling for initial aggressiveness [8], [9]. Experimental work suggests that playing violent video games is a causal risk factor for increased aggression [10]–[12]. These findings were corroborated in a comprehensive meta-analysis by Anderson and colleagues that yielded a significant average violent video game effect size for aggressive behavior [13]. The effect was reliable across the different study designs and publication bias did not account for these findings. These findings were replicated in a more recent meta-analysis that summarized all violent video game studies that appeared since 2009 [14]. As in the Anderson et al. meta-analysis, there was a significant association between violent video game exposure and aggressive outcomes.

However, there are also studies that failed to find that violent video games cause aggression [15], [16], and even some meta-analyses, using a smaller set of available studies compared to the Anderson et al. meta-analysis, suggest that the overall effect is close to zero [17], [18]. Thus, despite hundreds of studies, there is continuing scientific debate about the extent to which violent video games increase aggression.

Likewise, many lay people are concerned that playing violent video games increases aggression, whereas this stance is disputed by others. For instance, a recent representative sample of 2,278 American adults showed that 58% somewhat agreed or strongly agreed that there is a link between playing violent video games and teenagers showing violent behavior, whereas 32% somewhat disagreed or strongly disagreed. The remaining 10% were unsure [19]. It thus appears that most people believe that playing violent video games increases aggression (believers), but this stance is questioned by a considerable number of others (skeptics).

The present research examined believers' and skeptics' responses to scientific studies examining the link between violent video game play and aggression. Abundant research has shown that people are more willing to search for congruent information and abstain from seeking out incongruent information [20]. That is, believers should be more likely to read scientific studies showing an effect of violent video games on aggression, whereas skeptics are more likely to seek out scientific studies showing non-significant findings. As a consequence, it is to be expected that both sides feel validated after learning new facts that are congruent with their beliefs.

But what happens if one is confronted with inconclusive evidence? Many news media reports operate according to a fairness doctrine, in that both sides of a debate are presented even when the overall evidence clearly supports one view over the other [21]. Likewise, news reports about the effects of video game violence often provide a balanced coverage of both sides [22], [23]. Do people become more or less certain of their initial views after learning about mixed evidence? Based on previous research into biased assimilation and attitude polarization, it was predicted that believers would evaluate a study showing increased aggression after violent video game play more favorably than a study showing no effect. In contrast, skeptics would evaluate the no-effect study more favorably than the positive effect study (*biased assimilation*). As a consequence, both believers and skeptics should feel validated and report to become more convinced of their initial view (*attitude polarization*). Hence, even when learning about mixed evidence, the perceived gap between believers' and skeptics' views should increase.

Biased assimilation is the tendency to evaluate belief-consistent information more positively than belief-inconsistent information. In a seminal study, participants were first asked whether the death penalty is an effective deterrent against murder [24]. Both proponents and opponents of this view received short reports of two studies, one supporting the deterrent efficacy of the death penalty and one supporting the opposite viewpoint. Proponents of the death penalty evaluated the pro-deterrence study more favorably than the anti-deterrence study, whereas opponents of the death penalty were in favor of the methodology of the anti-deterrence study. This basic finding of biased assimilation has been replicated in different contexts, such as the safety of nuclear power [25], theories regarding the JFK assassination [26], and stereotypes associated with homosexuality [27].

After evaluating the two opposing studies, Lord and colleagues asked their participants about the perceived changes in their attitudes toward capital punishment. Results showed that both proponents and opponents became more polarized in their opinions (attitude polarization): When they were asked to compare their current attitude toward capital punishment with the attitude they had at the beginning of the experiment, proponents reported that they were even more in favor of capital punishment, whereas opponents reported that they were even more against capital punishment. Note that Lord and colleagues did not ask their participants to indicate their actual attitude toward capital punishment after reading the essays, so actual attitude change was not assessed. This is important insofar as subsequent research has replicated attitude polarization for reported attitude change [24], [28], but polarization for actual attitude change could not be observed [28]–[30]. If anything, actual attitude change was in the direction of depolarization.

### The Present Research

The present research employed a similar design as these previous investigations. Participants were asked to what extent they believe that playing violent video games causes aggression (initial belief). Afterwards, they were given summaries of two fictitious studies about the effects of violent video games. Whereas one study apparently found support for increased aggression after violent video game play (positive effect study), the other study found no significant association (no-effect study). After evaluating both studies, participants were asked to indicate their perceived attitude change as well as their belief toward the effect of violent video games on aggression after learning about these two studies (final belief). It was predicted that believers would evaluate the positive effect study more favorably than the no-effect study, whereas the opposite pattern should be observed for skeptics. Moreover, this biased assimilation should lead to differences in reported attitude change, in that believers report to become even more convinced that violent video games increase aggression, whereas skeptics report to become even less convinced. In terms of actual attitude change, no attitude polarization was expected.

As a further aim, the present study examined whether regular players of violent video games would be less likely to believe that violent video games increase aggression than non-regular players. Most, if not all, people are motivated to perceive themselves in a positive light and to make their beliefs consistent with their behaviors. Because behaviors are often more difficult to change than beliefs, people tend to alter their beliefs to make them consistent with discrepant behavior. There is wide consensus that aggression is an unwanted behavior. Thus, if players of violent video games learn that their behavior makes aggressive, their self is threatened. This reasoning holds that regular, more than non-regular, players tend to deny the existence of the negative consequences of violent video game play. In fact, people scrutinize information that is not compatible with a positive self-view, whereas they tend to accept information that does not threaten the self at face value [31]–[33]. Moreover, recent research [34] found that people who identify with the group of gamers are more likely than lowly identified people to devalue scientific evidence showing detrimental effects of violent video game play. Hence, it is further assumed that regular players of violent video games evaluate the positive effect study more favorably and the no-effect study less favorably than non-regular players. As noted above, biased information processing is associated with perceived attitude change. Thus, both regular and non-regular players of violent video games should report to be more convinced of their beliefs after receiving additional information than before.

Finally, previous research suggests [35], [36] that males are more likely than females to play violent video games. Thus, it was further hypothesized that males more than females believe that violent video games do not increase aggression and that biased assimilation and perceived attitude polarization differ for both sexes, in that males evaluate the positive effect study more favorably and the no-effect study less favorably than do females. Moreover, whereas males should report to become more convinced that violent video games do not increase aggression, females should report to become more convinced that violent video games increase aggression.

In Austria, it is not necessary to get explicit ethical approval if the study conforms to the guidelines of the German Psychological Society. Because this is the case for the current study, I got tacit approval from our ethics review board. That is, approval was waived. At the beginning of the study, participants read detailed instructions regarding ethical guidelines (i.e., that the data are analyzed anonymously and that they are free to abstain from participation in the study or to withdraw consent to participate at any time without reprisal). This instruction page serves as documentation of participants' informed consent. Our ethics committees/IRBs approve this consent procedure.

## Methods

Participants were 666 students who were recruited via a university mailing list. Because psychology students may have learned about the relation between violent video games and aggression in class, only non-psychology students received the invitation to participate. Attentive participation was verified with an item manipulation check [37], which was placed among the dependent measures. The item read: “We want to ensure that you read the survey attentively. Therefore, please skip this item.” Four individuals responded to this item and thus failed the item manipulation check. These participants were excluded from further analyses. However, the pattern of findings was virtually unchanged when these participants were included. The final sample included 662 participants (390 female, 272 male; mean age: 23.9 years, *SD* = 5.8).

At the onset, participants learned that the study was about the effects of violent video game play. About half of the participants were then asked to name their three favorite video games, to estimate the number of hours per week spent playing each video game, and to rate how violent the content of each video game was. For each video game, the amount of playing time was multiplied by violent content. These three violent video game exposure scores were averaged to provide an overall index of violent video game exposure. This approach has been successfully employed in previous video game research [6], [10], [38], [39]. For the remaining participants, violent video game exposure was measured at the end of the survey. However, this order variable did not affect the pattern of findings and is thus not considered further.

Then, participants indicated their initial belief toward whether violent video games increase aggression. To this end, participants responded to a single Likert-type item. The anchors were: −5 (*strongly decrease aggression*), 0 (*no effect*), +5 (*strongly increase aggression*). There were 397 participants who thought that violent video games would increase aggression, 142 participants thought violent video games had no effect on aggression, and 123 participants who thought that violent video games would decrease aggression. For the following analyses, the latter two categories were combined, so that participants who thought that violent video games would increase aggression (believers) were compared with participants who thought that violent video games would not increase aggression (skeptics). Because no study had to be evaluated that allegedly shows that violent video games *de*crease aggression, the pieces of new information employed in the present study (i.e., the positive effect study allegedly showing that violent video games do increase aggression and the no-effect study allegedly showing that violent video games do not increase aggression) were inconsistent and consistent with the initial beliefs for both participants who thought that violent video games had no effect on aggression and participants who thought that violent video games would decrease aggression.

Afterwards, participants were presented with two opposing summaries of fictitious studies on the effects of violent video play. These two studies allegedly appeared in scientific journals in 2012. After each summary, participants indicated to what extent the study was convincing and replicable in future studies, respectively. The scale for both items was from 1 (*not at all*) to 7 (*very much*). The positive effect summary read:

“The authors demonstrated that playing violent video games is associated with increased aggression. This effect was observed for adults as well as children and teenagers. In an experiment with 273 participants, participants who had played a violent video game were more aggressive afterwards than participants who had played a neutral video game. It thus appears that video games increase aggression in the short-term. It is unclear, however, whether violent video games affect real-life aggression in the long-term.”

The no-effect summary read:

“The authors noted that previous research into the effects of violent video games showed mixed findings and the interpretation of those findings is uncertain. In an online-study, 465 teenagers indicated their exposure to violent video games and rated their own aggressiveness. Results revealed no relation between violent video game exposure and physical aggression. Moreover, there were no effects of violent video game play on aggressive behavior six months later. However, no real behavior was assessed, so the researchers had to rely on participants' self-reports.”

Participants were randomly assigned to read the positive effect study either first or second. However, study order did not qualify any of the main findings and is thus not considered further. Afterwards, reported attitude change was assessed by asking participants whether their belief toward the increasing effect of violent video game play on aggression had changed after reading the two summaries. The anchors were: −5 (*less convinced*), 0 (*unchanged*), +5 (*more convinced*). Participants also indicated their final belief toward whether violent video games increase aggression, using the same anchors as before. Finally, participants were thanked and debriefed.

## Results

### Biased assimilation

**Figure 1:**
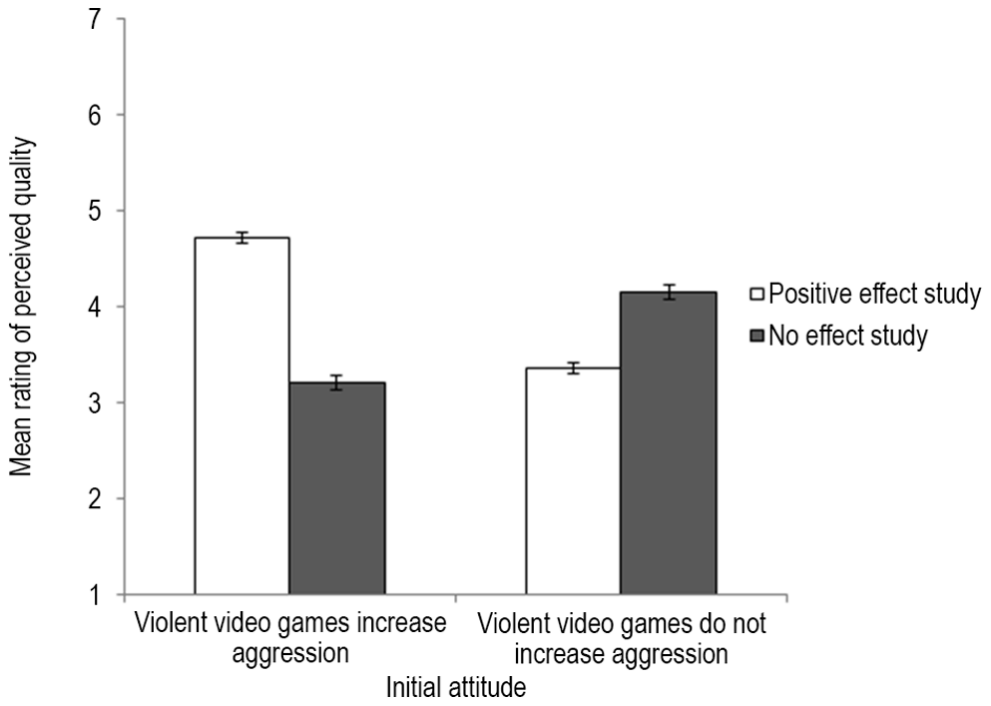
Mean ratings of perceived quality as a function of initial attitude and study effect. Error bars depict two standard errors.

Ratings of how convincing and replicable the study is were highly correlated and were, thus, averaged into a general perceived quality index (α = .74 for positive effect study, α = .73 for no-effect study). This general perceived quality index was analyzed in a 2 (initial belief: believers vs. skeptics)×2 (study: positive effect vs. no effect) analysis of variance (ANOVA), with study as a within-groups factor. Results revealed the predicted significant interaction between initial belief and study, *F*(1, 660) = 272.03, *p*<.001, η^2^ = .29 (Figure 1). Believers evaluated the positive effect study (*M* = 4.71, *SD* = 1.23) more favorably than the no-effect study (*M* = 3.21, *SD* = 1.13), *F*(1, 396) = 289.76, *p*<.001, η^2^ = .42, whereas skeptics evaluated the no-effect study (*M* = 4.15, *SD* = 1.31) more favorably than the positive effect study (*M* = 3.36, *SD* = 1.21), *F*(1, 264) = 54.39, *p*<.001, η^2^ = .17. Thus, there was strong evidence for biased assimilation.

Splitting the initial belief variable into believers and skeptics is the standard procedure in the literature. It also tellingly illustrates the point that people with opposing beliefs differ in the way how they evaluate novel, conflicting information. Nevertheless, the use of a continuous measure rather than dichotomizing the beliefs increases statistical power. In fact, the increase in power should enable participant sex and video-game play to be included in the analyses. As in past research [24], [27], ratings of the positive effect study and no-effect study were combined into a difference score by subtracting the ratings of the no-effect study from the ratings of the positive effect study. In a multiple regression, this difference score was regressed on the continuous measure of initial beliefs, participant sex (dummy coded: female = 1, male = 2), and the extent to which participants reported to play violent video games. The overall regression was significant, *F*(3, 658) = 104.29, *p*<.001. Most importantly, initial belief significantly predicted the difference score, β = .50, *t*(658) = 13.44, *p*<.001. Moreover, participant sex received a significant regression weight, β = −.07, *t*(658) = 2.01, *p* = .045, whereas violent video game play marginally significantly predicted the difference score, β = −.07, *t*(658) = 1.93, *p* = .054. In sum, the regression analysis replicated the main finding that initial belief had an effect on biased assimilation, while controlling for participant sex and the extent to which participants reported to play violent video games. Participant sex and violent video game play effects will be addressed more thoroughly below.

### Reported attitude change

Attitude assimilation was observed for reported attitude change: believers reported becoming more convinced that violent video games increase aggression (*M* = +0.12, *SD* = 0.92), whereas skeptics reported becoming less convinced (*M* = −0.20, *SD* = 1.20), *F*(1, 660) = 14.26, *p*<.001, η^2^ = .02. That is, attitude assimilation was observed for reported attitude change.

This finding was replicated in a multiple regression where reported attitude change was regressed on the continuous measure of initial beliefs, participant sex, and the extent to which participants reported to play violent video games. The overall regression was significant, *F*(3, 658) = 9.50, *p*<.001. Initial belief significantly predicted reported attitude change, β = .15, *t*(658) = 3.40, *p* = .001, whereas participant sex, β = −.03, *t*(658) = 0.78, *p* = .435, and violent video game play, β = −.06, *t*(658) = 1.40, *p* = .162, received non-significant regression weights. Participant's differential evaluations of the studies (biased assimilation) were significantly correlated with reported attitude change, *r*(662) = .19, *p*<.001.

### Actual attitude change

**Figure 2:**
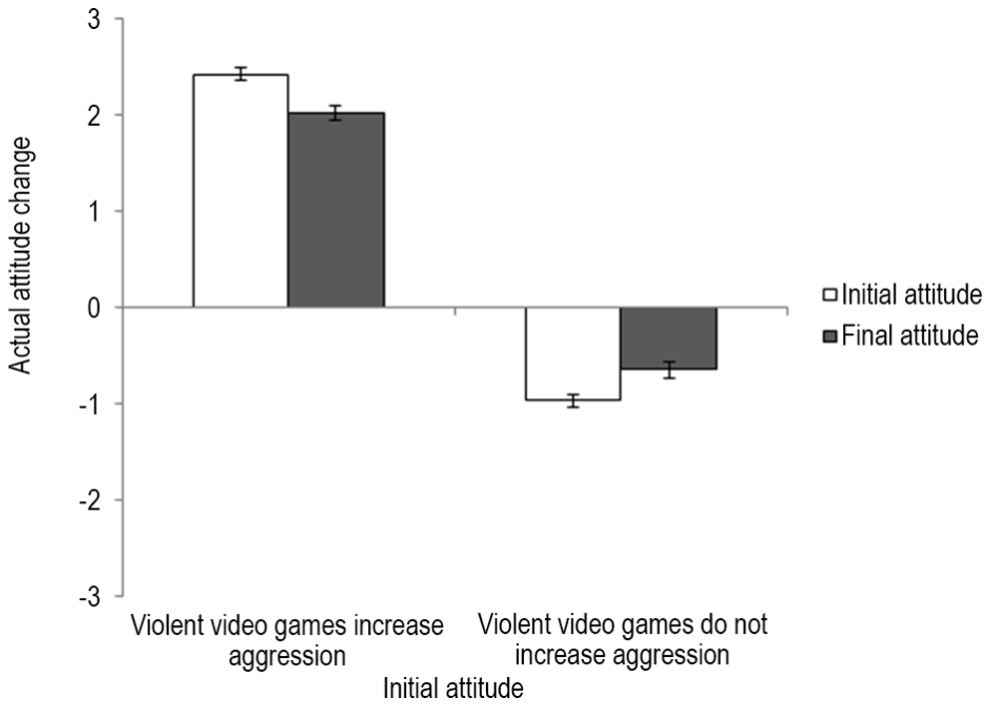
Mean actual attitude change as a function of initial attitude. Error bars depict two standard errors.

In the following, it was examined whether there was attitude polarization for actual beliefs. That is, believers should become more convinced that violent video games increase aggression after reading the summaries, whereas skeptics should become more convinced that violent video games do not increase aggression. To test this hypothesis, a 2 (initial belief: believers vs. skeptics)×2 (belief: initial vs. final) ANOVA, with belief as a within-groups factor, was performed on the data. Results revealed a significant interaction between initial belief and belief, *F*(1, 660) = 79.70, *p*<.001, η^2^ = .11 (Figure 2). However, contrary to attitude polarization, believers became less convinced that violent video games increase aggression after reading the summaries (*M* = 2.02, *SD* = 1.47) than before (*M* = 2.42, *SD* = 1.21), *F*(1, 396) = 62.26, *p*<.001, η^2^ = .14, whereas skeptics became less convinced that violent video games do not increase aggression after reading the summaries (*M* = −0.65, *SD* = 1.34) than before (*M* = −0.97, *SD* = 1.30), *F*(1, 264) = 25.61, *p*<.001, η^2^ = .09.

For the multiple regression analysis, initial beliefs were subtracted from final beliefs. The overall regression was significant, *F*(3, 658) = 48.54, *p*<.001. Initial belief significantly predicted actual attitude change, β = −.48, *t*(658) = 11.87, *p*<.001. Whereas participant sex was not associated with actual attitude change, β = −.03, *t*(658) = 0.78, *p* = .438, violent video game play did receive a significant regression weight, β = −.17, *t*(658) = 4.18, *p*<.001. Biased assimilation was not significantly correlated with actual attitude change, *r*(662) = .02, *p* = .658. Moreover, reported attitude change was not related to actual attitude change, *r*(662) = .02, *p* = .624. Thus, biased assimilation may underlie the effect of initial attitude on reported attitude change, but could not mediate the effect on actual attitude change.

### Mediational analysis

The mediation analysis examined whether participant's differential evaluations of the studies (biased assimilation) would mediate the effect of initial belief on reported attitude change. In fact, a bootstrapping analysis based on 1,000 bootstraps confirmed that the indirect effect was significantly different from 0 (*p*<.05, 95% confidence interval [−0.30, −0.07]), indicating that biased assimilation accounted for the finding that believers and skeptics reported to become more convinced of their initial attitude.

### Violent video game play

Violent video game play was related to initial belief, *r*(662) = −.46, *p*<.001, rating of the positive effect study, *r*(662) = −.25, *p*<.001, rating of the negative effect study, *r*(662) = .27, *p*<.001, and reported attitude change, *r*(662) = −.15, *p*<.001. That is, the more participants reported to play violent video games, the less they were convinced that violent video games increase aggression, the more negatively the positive effect study was evaluated, the more favorably the no-effect study was evaluated, and the less they reported becoming convinced that violent video games increase aggression. In contrast, violent video game play was not related to actual attitude change, *r*(662) = .04, *p* = .324.

### Sex of participant

Male participants (*M* = 9.60, *SD* = 9.20) reported to play more violent video games than female participants (*M* = 2.86, *SD* = 6.41), *F*(1, 660) = 123.54, *p*<.001, η^2^ = .16. As predicted, female participants (*M* = +1.69, *SD* = 1.98) were more likely to be convinced that violent video games increase aggression than male participants (*M* = +0.16, *SD* = 1.88), *F*(1, 660) = 99.63, *p*<.001, η^2^ = .13. They (*M* = 4.42, *SD* = 1.31) evaluated the positive effect study more favorably, *F*(1, 660) = 31.92, *p*<.001, η^2^ = .05, and the no-effect study less favorably (*M* = 3.34, *SD* = 1.20), *F*(1, 660) = 34.22, *p*<.001, η^2^ = .05, than did the male participants (*M* = 3.81, *SD* = 1.42; *M* = 3.93, *SD* = 1.33, respectively). Female participants (*M* = +0.09, *SD* = 1.02) also reported becoming more convinced that violent video games increase aggression, whereas male participants reported becoming less convinced (*M* = −0.15, *SD* = 1.09), *F*(1, 660) = 8.46, *p* = .004, η^2^ = .01. In terms of actual attitude change, however, female participants (*M* = −0.17, *SD* = 1.17) became less convinced that violent video games would increase aggression after reading the studies than before, whereas male's actual attitudes were almost unchanged (*M* = −0.01, *SD* = 0.93), *F*(1, 660) = 3.84, *p* = .050, η^2^ = .01.

When controlling for violent video game exposure, the sex differences in terms of initial belief, *F*(1, 659) = 33.40, *p*<.001, η^2^ = .05, evaluation of the positive effect study, *F*(1, 659) = 11.45, *p* = .001, η^2^ = .02, and evaluation of the negative effect study, *F*(1, 659) = 11.56, *p* = .001, η^2^ = .02, remained significant, but were considerably reduced. In all analyses, the effect of violent video game exposure was significant, all Fs>27.29, all ps<.001, all ηs^2^>.04. That is, differences in the amount of violent video game play accounts for some of the sex differences in terms of initial beliefs and evaluation of the studies. Moreover, for both reported attitude change and actual attitude change, the sex difference was no longer significant when controlling for violent video game exposure, both Fs<2.91, both ps>.089, both ηs^2^<.01.

## Discussion

The notion that violent video games cause aggression is a topic of hot debate. The present research also shows that there is considerable disagreement on this issue. Out of 662 participants, 397 thought that violent video games would increase aggression, whereas the remaining 265 participants did not believe so. In line with previous research into biased assimilation [24]–[30], whether participants believed or did not believe that violent video games increase aggression strongly affected how they evaluated studies either supporting or failing to support this claim. Whereas believers evaluated a study showing an effect more favorably than a study showing no effect, the exact opposite was observed for skeptics. As a consequence, both believers and skeptics reported to become more convinced of their initial attitudes. In sum, both sides felt to be supported from objectively mixed and inconclusive evidence, which then increases the gap.

It is noteworthy that females much more than males believe that violent video games increase aggression. Whereas 297 out of 390 female participants (76%) indicated that they thought violent video games would increase aggression, only 100 out of 272 male participants (37%) did so. Part of this substantial sex difference was accounted for by the fact that male participants reported to be more likely to play violent video games than female participants. In general, participants who regularly play violent video games were less likely to believe that doing so makes the players aggressive than participants who do not regularly play violent video games. Because this finding is based on a correlational design, one does not know whether players of violent video games are motivated to defend their own behavior or whether people play violent video games *because* they are convinced that playing violent video games is harmless. Future experimental work may test these competing accounts.

The present study addressed participant's responses to new information about possible negative effects of violent video game play (i.e., increased aggression). But video game play may also positively affect social outcomes [14], [40]. For example, playing prosocial video games increases helping behavior [41], [42] and decreases aggressive outcomes [43], [44]. Likewise, playing cooperative team-player (relative to a single-player) video games increases cooperative behavior [45]–[47]. It would be interesting to examine whether players of prosocial and cooperative team-player video games are more likely than non-players to believe that playing these video games positively affect social behavior. Such a finding would constitute further evidence that video game players are motivated to perceive themselves in a positive light.

Some limitations of the present study have to be acknowledged. Biased assimilation was assessed by two items, whereas only one measure was employed to assess initial and final beliefs. Moreover, participants only responded to two conflicting pieces of evidence. Note that this is the standard procedure in the biased assimilation literature [24]–[29]. Nevertheless, future research may employ multiple pro-effect studies and no-effect studies that have to be evaluated on multiple measures. This would greatly improve the generalizability of the present work and would be also less prone to measurement error. In this vein, it is noteworthy that the two summaries of the studies varied on multiple dimensions (other than suggesting that violent video games do or do not increase aggression). However, because both sides of the debate assimilated the new information to their existing beliefs, one can be relatively sure that the present findings actually reflect confirmatory information processing. Future work would be also welcome that employs a non-student sample. It is conceivable, for example, that a less intelligent audience might be less able or less willing to counter-argue new inconsistent information. Finally, no suspicion check was employed so the extent to which demand effects contribute to the present findings is unknown.

Future research may address some behavioral implications of increased perceived attitude change after learning mixed video game evidence. For instance, people's future amount of violent video game play may be affected by their perceived attitude change. Interestingly, the increased gap was perceived but not real. In fact, the actual final beliefs of believers and skeptics were more similar than the initial beliefs (although initial believers were still much more likely to believe that violent video games increase aggression than initial skeptics)—a pattern of findings that is consistent with a regression toward the mean interpretation.

Because of the differences in terms of how evidence on the relation between violent video games and aggression is interpreted, it may not suffice when both sides of the argument know the most important pertinent facts. As shown in many studies, people are very able to maintain their existing beliefs by counterarguing information that does not fit their preexisting opinions [48], [49]: rather than facts itself but their interpretations affect people's beliefs. Both believers and skeptics could accurately perceive the same facts but make different interpretations of their meanings. For instance, whereas they may agree on the average estimate observed in a meta-analytic analysis, they may disagree, however, in terms of whether this estimate is big enough to warrant societal concern or is so small that it hardly matters whether people play violent video games or not. Moreover, knowing more facts may even foster partisan-motivated interpretations. Those who know most about an issue appear to be most willing and able to interpret information in a biased way so that they can maintain their beliefs [50].

Of course, it is possible that people's beliefs can shift in response to learning about new facts. If new evidence clearly contradicts one's beliefs, people may abandon belief maintenance. For instance, following Japan's Fukushima Daiichi nuclear disaster, Chancellor Angela Merkel's coalition announced that Germany's nuclear power stations will be shut down by 2022. This constituted a reversal of their own policy, because just one year ago the government had overturned the phase-out plan of the previous government and agreed on a 12-year delay of the schedule. Likewise, if substantial evidence accumulates on whether violent video games increase aggression, some people may consider changing their beliefs.

But because most partisans will find a way how incongruent new evidence can be discredited, it is likely that the threshold is very high before this really happens. So debiasing techniques may be needed to decrease assimilation of new information. Previous research has shown that increasing the availability of counterexplanations [51], [52] or asking people to process new arguments from the opposite of their own perspective [53] successfully reduces biased confirmatory information processing. Fostering perspective-taking of the other side might decrease the gap between believers and skeptics of violent video game effects.
